# Balancing medical innovation and affordability in the new healthcare ecosystem in China: Review of pharmaceutical pricing and reimbursement policies

**DOI:** 10.1002/hcs2.76

**Published:** 2023-12-11

**Authors:** Vivian Chen, Wenbin Shao

**Affiliations:** ^1^ Institute for Hospital Management Tsinghua University Beijing China; ^2^ Commercial Solutions, IQVIA Greater China Shanghai China

**Keywords:** medical innovation, affordability, ecosystem evolution, China healthcare, pharmaceutical pricing and reimbursement, market access, biotech, value‐based pricing, medical insurance

## Abstract

The China Basic Medical Insurance Program was created in 1999 with three objectives: equal accessibility, affordability, and quality. Today, it has become the biggest medical insurance program in the world, covering 95% of China's population. Since 2015, China's healthcare ecosystem has been reshaped by increasing innovation, which has in turn been driven by regulatory reform, enhancement of research and development capability, and capital market development. There has also been improved regulatory efficiency to reduce lags in launching drugs. In 2022, nearly 20% of novel active substances launched globally were from China. China has also risen to become the second biggest contributor to innovation in terms of pipelines. Using a “fast‐follow” strategy, many locally developed innovative drugs can compete with products from multinational companies in their quality and pricing. However, China's pharmaceutical and biotechnology industry will continue to face challenges in pricing and reimbursement, as well as a shortened product lifecycle with rapid price erosion. The government has already accelerated the timeline for updating the drug reimbursement list and is willing to create a high‐quality medical insurance program. However, some obstacles are hard to overcome, including reimbursement for advanced therapies, limited funding and an increasing burden of disease due to an aging population. This article reviews the trajectory of medical innovation in China, including the challenges. Looking forward, balancing affordability and innovation will be critical for China to continue the trajectory of growth. The article also offers some suggestions for future policy reform, including optimizing reimbursement efficiency with a focus on high‐quality solutions, enhancing the value assessment framework, payer repositioning from “value buyer” to “strategic buyer”, and developing alternative market access pathways for innovative drugs.

## Graphical abstract

China has the biggest medical insurance program in the world with coverage of 95% population, with tremendous pressure on cost control. Since 2015, China's healthcare ecosystem has been reshaped by the rising China innovation, driven by regulatory reform, enhancement of R&D capability, and capital market development. How to balance affordability and innovation will be critical for China to continue the trajectory.

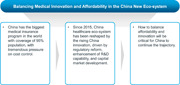

**Abbreviations**
BTD: breakthrough therapy designation
CAR‐T: chimeric antigen receptor T‐cell
CDE: Center for Drug Evaluation
CED: coverage with evidence development
CTA: clinical trial application
DIP: diagnosis intervention packet
DRG: diagnosis‐related group
EMA: European Medicines Agency
FDA: Food and Drug Administration
GBI: Global Business Intelligence
HCV: hepatitis C virus
HKEX: Hong Kong Stock Exchange
HTA: Health Technology Assessment
ICH: The International Council for Harmonization of Technical Requirements for Pharmaceuticals for Human Use
IND: Investigational New Drug
M&A: mergers and acquisitions
MNC: multinational corporation
NDA: New Drug Application
NHSA: National Healthcare Security Administration
NMPA: National Medical Products Administration
NRDL: National Reimbursement Drug List
PD‐1: programmed death protein‐1
PhIRDA: Pharmaceutical Innovation and Research Development Association
R&D: research and development
VBP: volume-based procurement

## 1 OVERVIEW OF THE NEW HEALTHCARE ECOSYSTEM IN CHINA

China's pharmaceutical industry has been transformed since 2015. This is shown in both the growing size of the pharmaceutical market and the growing degree of medicine innovation. Today, China is the second‐largest pharmaceutical market in the world [1], and is no longer dominated by generics. Innovative medicines have emerged quickly, and the rising level of innovation among local biotechnology companies has helped to shape China's innovation ecosystem. This trend is likely to continue over time.

Key underlying drivers were designed as a “three‐legged stool” to support earlier stages of innovation growth in China. They include:

(a) The government's innovation policy via the China Science and Technology Major Project, which has funded thousands of biotechnology startups since 2008 under the 5‐Year Plan [2];
(b) Pharmaceutical regulatory reform in 2015, designed to connect China to international communities; and
(c) Capital market reform by the Hong Kong Stock Exchange to allow the listing of pre‐profitable biotechnology companies in 2018 [3].

At the same time, native research and development (R&D) talent trained overseas has been returning to China, bringing knowledge and technologies to start their own biotechnology companies.

However, China's healthcare reform has also faced challenges to curb drug prices, control the growing funding pressure and improve affordability of drugs for patients [4]. The proliferation of innovation was encouraged by the government, but also created a challenge for it to balance different stakeholders during the emergence of a new market ecosystem with providers, services, and capital market.

To address the relationship between medical innovation and affordability requires good understanding of all the areas of policy that affect these areas. This paper identifies the three most important developments under the new healthcare ecosystem and provides a brief analysis of how China's pharmaceutical pricing and reimbursement policies have responded to these changes. Lastly, the paper offers some thoughts and a brief analysis of future prospects, highlighting three important developments.

### 1.1 Speed to market in a reformed drug regulatory system

The most important driver accelerating innovative drug development is the reform of the drug regulatory regime, initiated by China's National Medical Product Administration (NMPA), equivalent to the United States Food and Drug Administration (FDA). The NMPA was the most reform‐minded government agency in its early days, setting out a clear vision to advance and accelerate the drug review and approval system in China [5].

2015 was a pivotal year, when a new drug review and approval process started a sweeping reform initiated by the NMPA. It was also the year when China joined the International Council for Harmonization of Technical Requirements for Pharmaceuticals for Human Use (ICH) and introduced global standards for drug clinical trials and registration. The NMPA started to benchmark to global technical standards supported by ICH, and made a strong policy commitment to reduce the lags in drug launches and improve regulatory efficiency. This has been followed by the issue of several regulatory and policy changes to expedite procedures, as well as a revision of the Drug Administration Law and Administrative Measures for Drug Registration.

Delay in clinical trial applications was one of the main reasons for lags in launching drugs. To optimize the review and application process, the NMPA launched a 60‐day approval system for clinical trial application. IQVIA analysis shows that the average Investigational New Drug Application (IND) approval timeline decreased from 9–12 months to 2–3 months. Following a review and approval of revised marketing authorization processes, the revised Administrative Measures of Drug Registration was issued in 2020. This provides four major expediting registration procedures for breakthrough therapy designation approval, conditional approval, prioritized review, and procedures for special approval to improve time management and deliver essential innovative drugs to Chinese patients.

Encouraged by the “fast‐follow” strategy used for the process of regulatory policy reform, a similar strategy was adopted by the biotechnology industry to fast‐follow new targets and new mechanisms of action to shorten lags in drug launches. Collective efforts from the regulator and the industry have significantly shortened the new drug development timeline. This has led to a huge improvement in efficiency, speed to market, and R&D productivity.

As a result of these reforms, the difference in the time required to launch new drugs between China and the United States has dropped on average from 5–7 years to 1–2 years. For example, in 2010, the average lag in launch of oncology drugs between China and the United States was 7 years. By 2020, this had shortened to about 1 year. For orphan drugs, it was 9 years in 2010, reducing to around 1.5 years in 2020 [6].

More innovative drugs are expected to be launched in China in the future, and it is anticipated that time‐lags will be further shortened or even removed. As local innovation keeps evolving, it is highly possible that in the future, new drugs might be launched in China first. The impact of this emerging trend of new drug development on the overall strategy of market access should be watched closely [7].

### 1.2 Growing influence of China's pharmaceutical market

In 2016, China surpassed Japan to become the second‐largest pharmaceutical market in the world. Today, China and the United States combined account for 50% of the global pharmaceutical market. China supplies 185 billion USD in sales, and the United States has some 620 billion USD. According to IQVIA Market Prognosis, by 2027 the combined market of China and the United States will exceed 60% of a total global drug market worth 1.8 trillion USD [8].

**Figure 1:**
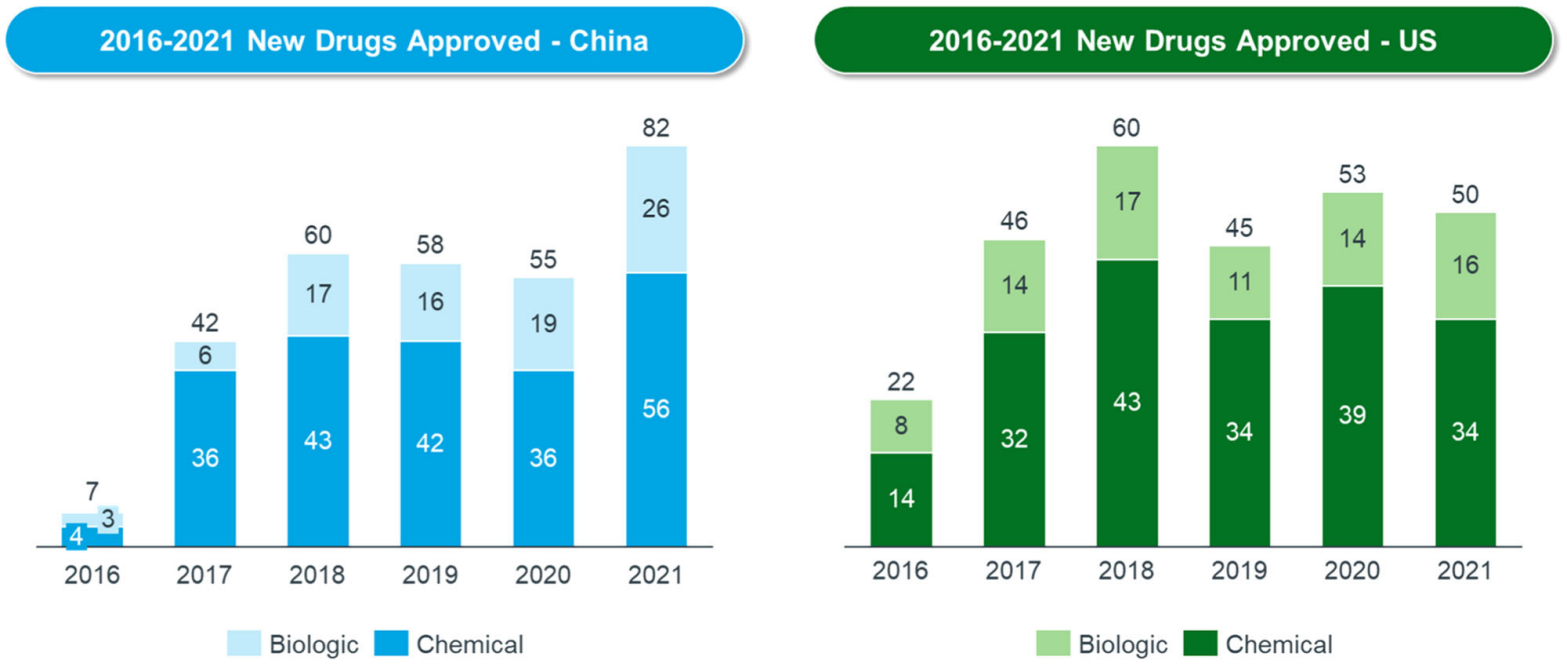
New drugs approved in China versus United States 2016–2021. *Source*: GBI Source data: https://source.gbihealth.com.cn/and FDA data: https://www.fda.gov/.

At the same time, new drug approvals have increased fast. Since 2017, the NMPA has approved over 400 new drugs. Each year, the total number of new drugs approved in China has risen faster, to match the level of the United States (see Figure 1). The approval of a growing number of Category 1 drugs (China's definition of new drugs) reflects the increasing degree of innovation in China.

**Figure 2:**
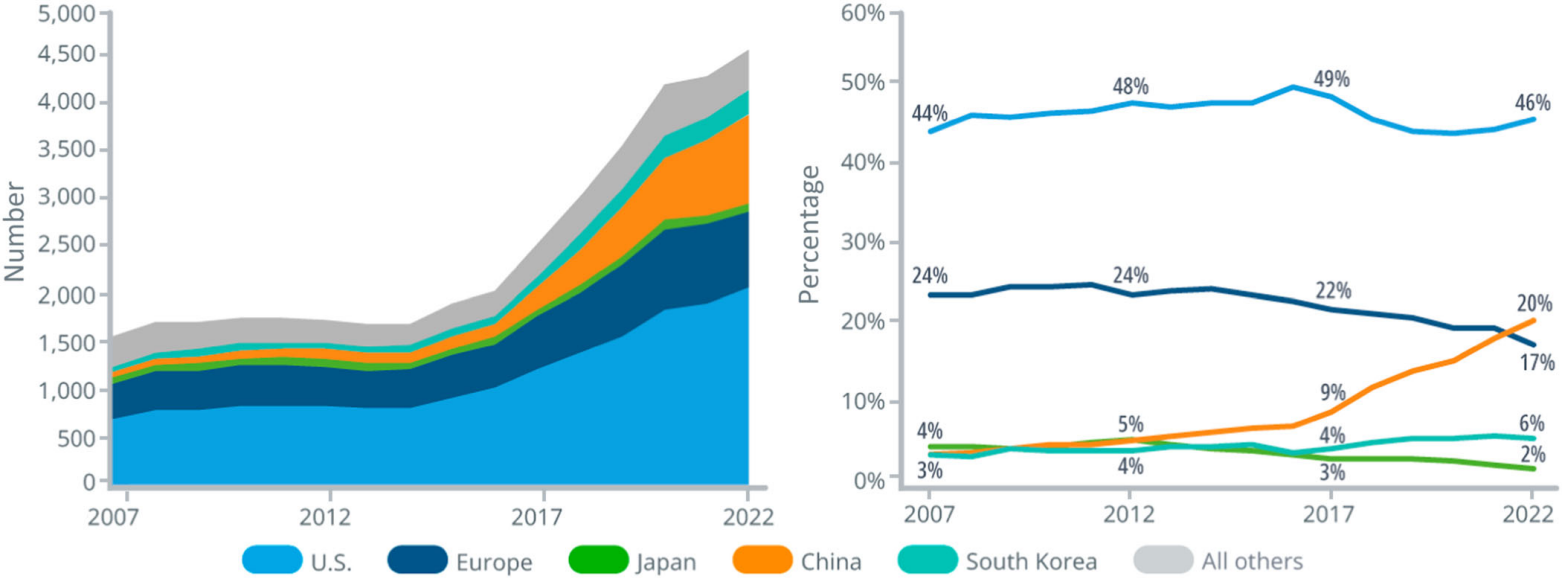
Number of drugs and countries' share of the emerging biopharma pipeline, Phase I to regulatory submission, based on company headquarter location, 2007–2022. *Source*: IQVIA Report ‐ Global Trends in R&D 2023, IQVIA Pipeline Intelligence, Dec. 2022; IQVIA Institute, Jan. 2023.

According to IQVIA, launches of novel active substances in China accounted for nearly 20% of global launches of these substances during 2021 and 2022. China has also moved up to become the second‐most innovative country in terms of pipelines. The IQVIA Report on Global Trends in R&D 2023 reported that China's share of global R&D pipelines has increased six‐fold over the past 15 years [9]. China is also the only market that is continuously rising compared with other major markets [9] (see Figure 2).

**Figure 3:**
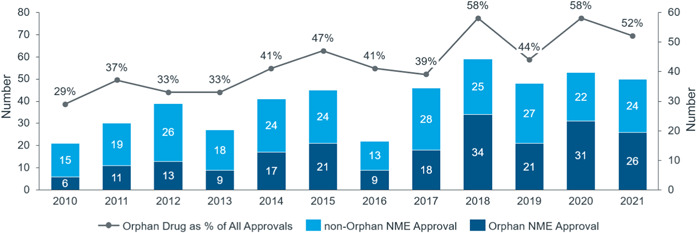
Orphan drugs as a proportion of new drug approvals. *Source*: US FDA 2020: https://www.fda.gov/drugs/.

Orphan drugs approved by the FDA account for 50% of new drugs approved each year [10] (see Figure 3). The relatively larger unmet needs in China mean that R&D pipelines there have a strong resemblance to those in the United States, with rising approvals of cancer drugs, orphan drugs, or drugs with rare disease designation. Overall, the proportion of biological drugs has increased significantly and is now close to 30% of all new drugs approved annually.

For example, among the new cancer drugs approved by the FDA over the past 5 years (2015–2020), 40% are also approved by the NMPA. In the period from 2004 to 2014, only 24% were approved by the NMPA [11]. However, despite the recent fast growth, there is a larger gap in “true innovation” between China and both the United States and European countries. This is due to China's short history of development of the pharmaceutical industry. On‐patent originators make up just over 10% of China's pharmaceutical market, and the proportion of innovative drugs from China is also very small in the international market, estimated at around just 3% [12].

### 1.3 Rise of local biotechnology and increasing market competition

For a long time, innovative drugs available in China were mostly developed by multinational companies. Treatments were expensive, and off‐patent drugs maintained higher prices even many years after patent expiration. However, China's biotechnology industry has benefited significantly from the drivers mentioned earlier. It experienced an unexpected boom from 2015 to 2020, followed by a slowdown. This rise in local biotechnology companies has shaped market dynamics profoundly.

Up to 2021, there were approximately 1600 biotechnology companies registered in China [13]. Today, there are about 76 companies listed on the Hong Kong Stock Exchange, Shanghai STAR, and Nasdaq, with a combined marketization value of around 118.5 billion USD. Beigene is the only leading biotechnology company to have achieved initial public offering (IPO) status on all three stock markets [14].

The number of clinical trial applications from local biotechnology companies has also increased quickly. Local companies accounted for 50% of clinical trial applications in 2020, of which 40% were on oncology drugs. This is a huge increase from 10 years ago when the local oncology drug pipeline was largely nonexistent.

To date, more than 80 new drugs in Category 1 have been approved by the NMPA, and the majority are from local biotechnology companies. The increase in Category 1 drugs shows that local biotechnology companies are able to translate compounds into real drugs at an earlier stage, highlighting increasing R&D capability. Representative breakthrough therapies, including HCV (hepatitis C virus), programmed death protein‐1 (PD‐1), and now gene and cell therapies, are now made by local companies and provided to Chinese patients. More importantly, the emerging local industry helps to reduce the prices of new drugs for expensive treatments, because of its contribution to increasing market competition [15].

Three stages of globalization for biotechnology companies have been identified. Success in each stage requires different sets of capabilities. Most firms start with the initial stage to secure a “license out”, then develop clinical trials and R&D centers in global markets (primarily the US and EU). Successful commercialization in the global market is the ultimate goal, but also the most challenging to achieve.

The globalization of China's biotechnology industry is still at an early stage. New drug development via cross‐border partnership in licensing agreements is the most common global activity. The latest GBI Health data shows 1286 transactions of license‐in and license‐out activities from 2016 to 2023, with a total value of 140 billion USD. However, in late 2022, total transactions of license‐in activities dropped by 63%, and those of license‐out activities increased by 25%. Such a big switch may show an increasing recognition of China's biotechnology R&D assets by global players actively optimizing pipelines of innovations across different markets, including China. However, the slowdown in licensing‐in agreements may also be partially explained by the negative impact of the COVID‐19 pandemic. As an example of licensing activity, in 2017, the Chinese company Legend formed a licensing agreement with Johnson & Johnson to codevelop the CAR‐T (chimeric antigen receptor T‐cell) therapy Carvykti to treat multiple myeloma [16]. A successful partnership enabled Carvykti to gain FDA approval in 2022 [17] and European Medicines Agency (EMA) market authorization in 2023 [18].

**Figure 4:**
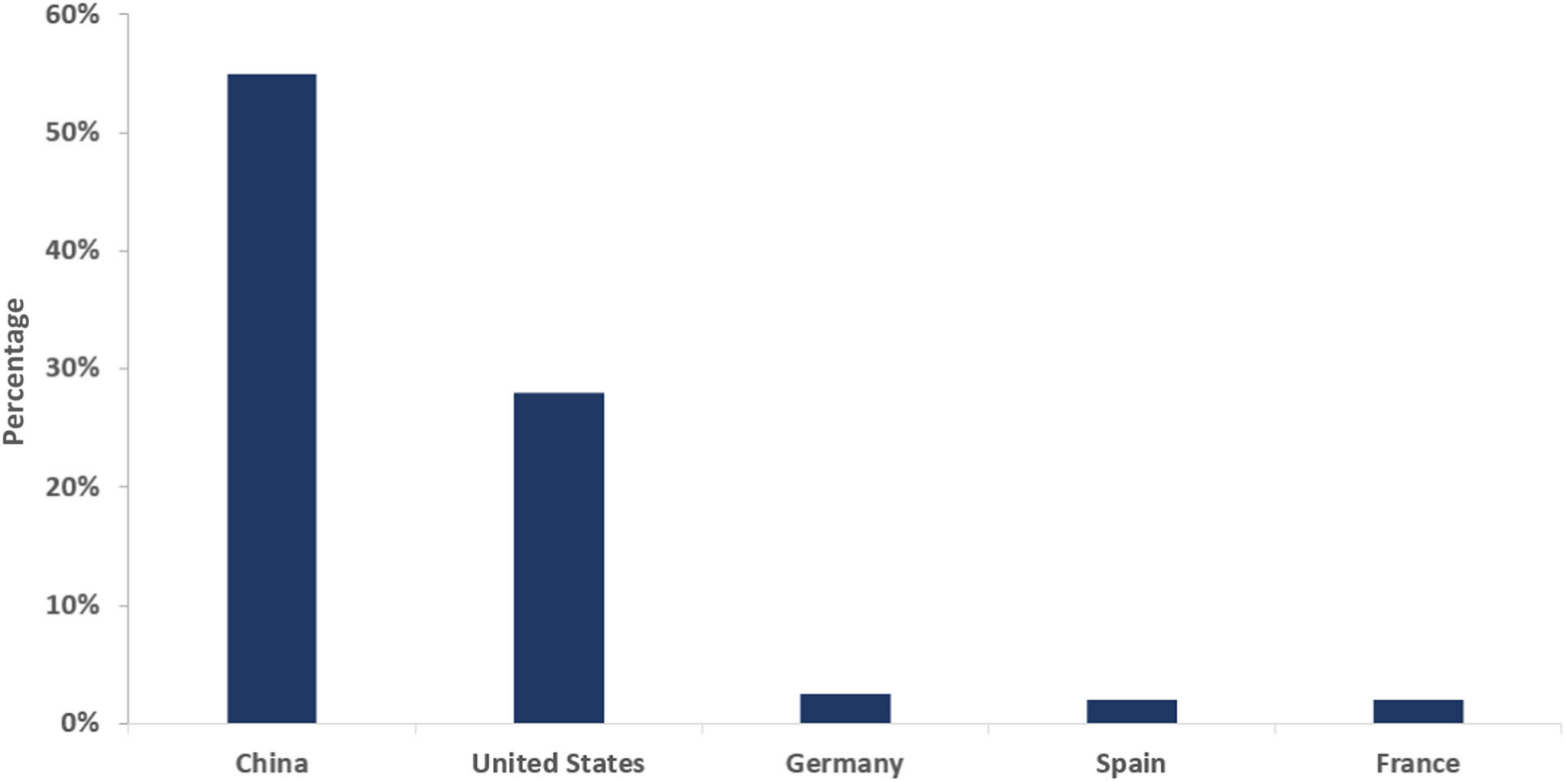
Percentage of clinical trials for CAR‐T therapies by country. *Source*: GlobalData, Pharma Intelligence Center, Deals Database: https://www.globaldata.com/data/.

The current R&D model in China is generally a “fast‐follow strategy”, because of the historically weak foundation of basic research. An increasing number of R&D activities is now conducted at global multicenters for clinical trial sites, but very few drugs have gained authorization from either the FDA or EMS to date. Beigene's Zanbrutinib was the first innovative drug from China to gain FDA approval, in October 2019. New drugs delivered by Chinese biotechnology firms are currently mainly “me‐too” products, with few classed as “me better” or first‐in‐class. However, China now dominates global CAR‐T therapy development. Of the 10 companies involved in the development of CAR‐T drugs, six are based in China [19]. On average, the number of newly identified CAR‐T therapies from Chinese developers has doubled every year since 2014 [20] (see Figure 4).

## 2 CHINA'S PHARMACEUTICAL PRICING AND REIMBURSEMENT POLICIES IN RESPONSE TO THE NEW ECOSYSTEM

How have China's pharmaceutical pricing and reimbursement policies responded to the changes in its healthcare and innovation ecosystem? What are the opportunities and challenges that lie ahead?

### 2.1 Establishing new leadership with expanded responsibility

In 2018, the newly‐created National Healthcare Security Administration (NHSA) was empowered with mandates from the central government and an enlarged scope of administrative responsibilities [21]. Three priorities were set under the new leadership. The first was to strengthen the world's largest medical insurance program by consolidating rural–urban resident and employment‐based insurance programs under the full supervision of the NHSA. The second was to address the importance of national equality and fairness of the government‐run program and increase centralized mandates to standardize benefit coverage for all, while phasing out local autonomy in benefit upgrade. This was designed to eliminate a potential risk from local provinces' flexibility to keep 15% of room to upgrade national drug reimbursement list (NRDL)‐based prices for local situations. The third was to redefine the primary role of basic medical insurance and limit its provision to basic medical benefit. This required reemphasizing the critical role of commercial insurance to fill the gap beyond basic medical coverage to meet the high costs of critical illness and provide patients with an option for more advanced therapies [22].

### 2.2 Moving to a new pricing and reimbursement strategy

Historically, spending on drugs was more than 30% of national healthcare spending [23]. higher than in most comparable countries. Over the past three decades, China has adopted several different drug pricing models. Before 2015, the government directly set a retail price ceiling, mainly based on product costs. Drugs were treated as public goods with a price cap [24]. Between 2015 and 2018, the government removed drug price control and drug prices were left to the market to determine on a temporary basis. However, despite these different pricing approaches, the high proportion of China's healthcare spending on drugs was never effectively tackled. No tangible outcomes were achieved because of the structural problem of the overall healthcare system.

To rationalize drug spending, associated with high drug prices and overuse at hospital level, the NHSA set out a new pricing strategy to shift from a cost‐plus approach to value‐based pricing. Since 2018, Health Technology Assessment (HTA)‐guided drug price negotiation has been adopted to support value‐based pricing, which brings China's pricing model closer to international practice. The NHSA's role has moved from a passive payer under the previous fee‐for‐service payment system to that of a more strategic purchaser, focusing on value and evidence [25].

Policy confidence to move forward with the new pricing strategy, known as a price–volume trade off, has been provided by China's ownership of the world's largest patient base. It has been further validated by recent drug market performance. Price reductions can always be compensated for by enlarged sales from broader market access. China is therefore still an attractive market for most drug companies despite cost control.

The NHSA has launched three nationwide strategic purchasing programs, affecting the current market and with far‐reaching ramifications for the future. The first program is a centralized hospital drug procurement program, designed to promote generic substitution for off‐patent drugs, and increase market competition to lower high drug prices and remove excessive mark‐ups by distribution channels. The second program is a national drug price negotiation program, designed to build up the capacity of value‐based pricing for newly approved patented drugs, by leveraging HTA assessment. The third program is the hospital payment system, reformed from fee‐for‐service to use diagnosis‐related groups (DRGs) and diagnosis‐intervention packets (DIPs). These currently cover about 35%–40% of inpatient treatment items at hospital level. The aim is to streamline drug use at hospital level via payment system reform. Average price reduction for both strategic purchases is around 50%, and price reductions can be as high as 90% for some more expensive prescription drugs. Since 2018, the total savings under these first two programs has been around 960 billion RMB [26].

In exchange for the larger price reduction driven by the payer's strategic purchasing to control increasing costs, several major positive measures have been taken by the NHSA to offset the revenue losses resulting from price reduction. First, the NHSA made a commitment to the R&D industry to speed up market access for reimbursement updates driven by drug regulatory reform. It also promised more frequent updates of the NRDL. Since 2017, the NDRL update timeline has shortened from 5 years on average to once per year. The latest NRDL Guideline, issued in 2020, set out a formal commitment to an annual update of the NRDL [27].

Second, to support annual NRDL update and price negotiation, the NHSA also committed to formally adopting HTA assessment for new drug price setting. Using years of HTA research, this process has now been applied for new drug price setting for 7 years, or seven rounds of drug price negotiation. HTA dossier submission is required for any drug company to enter price negotiations [28]. In the new ecosystem, the HTA provides value for payers to further develop a high‐quality, evidence‐based, more scientific, and fair pricing decision‐making mechanism. Over time, the process and actions related to price negotiation, organizational capacity, HTA capacity, and harmonization of cost effectiveness analysis versus budget impact analysis in decision‐making will become more transparent and efficient.

**Figure 5:**
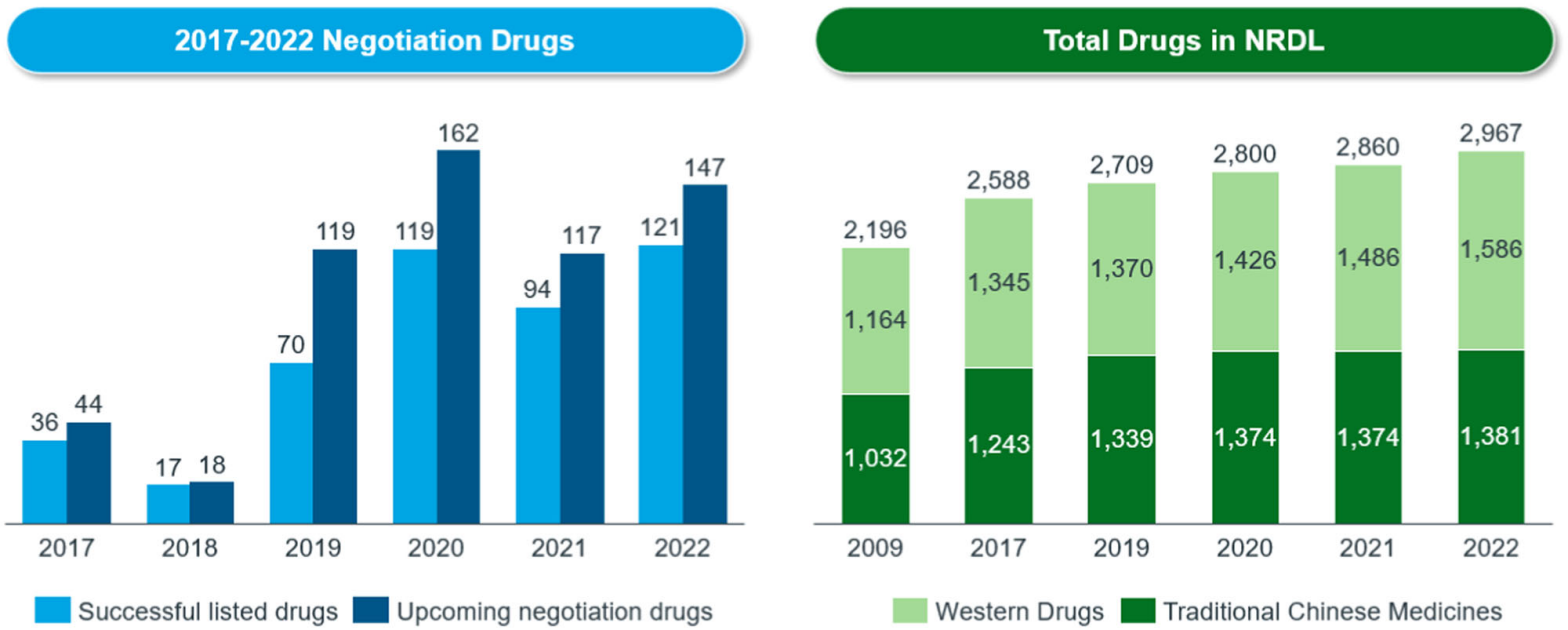
Number of drugs included in the NRDL. *Source*: government official news: https://www.gov.cn.

Third, to accommodate fast reimbursement of new therapies, it is important to improve the efficiency of budget allocation. New criteria on the inclusion and exclusion of reimbursable drugs are defined in the Guideline. The 2019 NRDL update delisted 154 drugs because they were providing less value. However, each year, about 92 new drugs are added to the NRDL. At present, around 3000 drugs are covered under the national drug reimbursement scheme, including some high‐value drugs such as HCV, PD‐1 and some orphan drugs (see Figure 5). More than 60 orphan drugs are listed under the NRDL.

### 2.3 Establishing price management across product lifecycles with increasingly transparent procedures and predictable rules

The concept of price management across the product lifecycle is relatively new in China, and was not aligned with the new pricing policy until 2019. The 2023 NRDL Adjustment Guideline sets out the concept, including concrete rules. It spells out different pricing rules and solutions applied to different stages of the product lifecycle. The new guideline, especially the pricing rule for multi‐indication drugs, has been welcomed by the industry. It has helped to improve the transparency and predictability of the new pricing policy, which is critical to enable the industry to comprehend, plan and prepare [29].

There are some important advantages to China from this approach. First, before inclusion in the NRDL, the price of new drugs is determined by companies themselves. For drugs included in the NRDL, the price is set via negotiation between payer and manufacturer. In general, a larger price reduction is expected in exchange for nationwide reimbursement status. In practice, negotiated pricing is only valid for 2 years. When the 2‐year pricing contract expires, prices are renegotiated. When drugs have been available for several years, are near maturity, or have gone through at least two rounds of pricing contract renewal, there are minimal further price cuts.

Similarly, drugs that have been available for at least 8 years and have little impact on the insurance budget are not included in subsequent price negotiation. Their status changes from a high price to less expensive category within the NRDL, avoiding further price cuts. Finally, when a patent expires, there will be a larger price cut via generic competition or centralized competitive procurement.

The high speed of approval and larger volume of new drugs approved by the Center of Drug Evaluation in recent years means that the timeline for market access for new drugs before an NRDL update is now shorter, along with the new drug product lifecycle. Market performance of “me‐too” drugs at peak sales appears to be lower than for similar drugs in other mature markets. The IQVIA hospital audit database suggests that in 2022, fewer than 10 drugs achieved annual sales of 4 billion RMB.

### 2.4 An evolving innovation ecosystem and pricing and reimbursement policies

Pricing and reimbursement policies do not always passively react to the changing innovation ecosystem. Regular NRDL update, price negotiation and volume‐based procurement have established a clear market access pathway for pricing and reimbursement for China's pharmaceutical industry. They also drive the direction of development of the innovation ecosystem. Innovations are encouraged to be earlier, faster and smarter to gain more time and sufficient evidence before negotiation and NRDL update. They also need to be true, meaningful, and economical. This is expected to drive more delisting from the NRDL.

**Figure 6:**
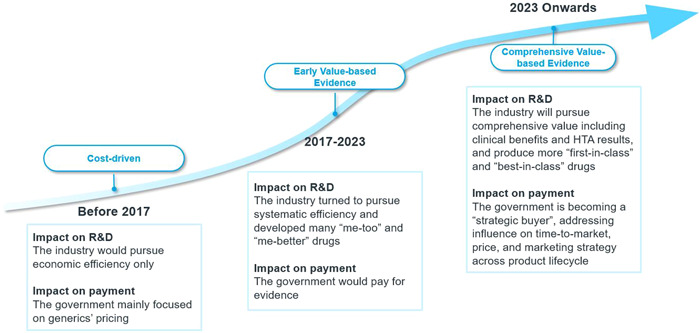
The evolution of China's innovation ecosystem. *Source*: IQVIA analysis.

We are therefore seeing China's pricing and reimbursement policies evolving from cost‐driven to balancing cost and value based on evidence (see Figure 6). The NHSA has set a higher goal in its new working plan released in June 2022. This asked for more detailed, comprehensive, and multicriterion evidence to be used when reviewing the value of pharmaceuticals. The score is weighted for efficacy, safety, economy, innovation, and equity, reflecting the NHSA's willingness to pay. A higher score means a higher reimbursement standard.

## 3 CHALLENGES AND PROSPECTS

### 3.1 Challenges facing the R&D industry

Despite the positive progress to date, there are still various difficulties and concerns for the R&D business. Speed to market remains a challenge because of difficulties getting hospital access in spite of regulatory and reimbursement reform. Larger price cuts for innovative drugs under price negotiation can be an impediment to drug innovation. The lack of a workable pricing solution for high‐cost advanced therapies could also discourage further investment in R&D [30].

### 3.2 Challenges facing the government payer

The payer is motivated to develop a high‐quality medical insurance program, but faces three major challenges. It needs to find a pricing solution that will address R&D complexity, budgetary pressure, and the high out‐of‐pocket payments required of patients. The main areas of R&D complexity are multi‐indication drugs, combined therapies, and precision medicines for curable diseases, such as gene and cell therapies. These incur high upfront costs and make pricing for innovative treatment complicated. Uncertainties associated with clinical efficacy and budget impact under conditional approval procedure contribute further to pricing complexity.

Second, economic affordability is an issue because of demographic changes from the aging population and changing disease profile, especially the high cancer incidence rate. This could lead to long‐term budgetary pressure for the payer. Third, the increase in patients' out‐of‐pocket payments is closely associated with lower benefit standards, constrained by the basic provision of the medical insurance program. This may be further aggravated because broader access to commercial insurance has not yet been achieved in China. For example, self‐pays for cancer drugs can be as high as 50% there [31].

It is therefore important for the payer to develop comprehensive solutions to balance medical innovation and affordability. Addressing these challenges will require improvements in funding efficiency, including allocating extra budget to the healthcare sector. They also required increased administrative capacity, and improvements in policy research, policy design, HTA efficiency, professional capacity, and coordination across agencies. Communication between the reimbursement agency and regulatory agency is becoming particularly important.

### 3.3 Some prospects

#### 3.3.1 Focus more on the quality of care

The main objectives of any country's medical insurance program are coverage, cost, and quality. China has so far achieved reasonably good outcomes for the first two. “High quality”, meaning better treatments, will be prioritized going forward. Many new treatments and breakthrough drugs are not yet available in China. Those that have been marketed in China often place a high financial burden on patients. Policymakers therefore need to find ways to lower drug and therapy costs, achieve high levels of coverage, and foster a healthy ecosystem for innovation.

#### 3.3.2 Establish extensive frameworks for value assessment

It is challenging for payers to evaluate the true value of innovations and new regulatory approvals. Value assessment needs careful consideration, above and beyond conventional cost‐effectiveness analysis. To enable socially viable patient access to high‐value drugs may require consideration of better clinical value in combination with other factors, such as unmet need, disease burden, level of market competition, and broader social equality and fairness. In some cases, the payer may need to create a comprehensive assessment framework to deal with innovative drug development and the regulatory system.

#### 3.3.3 From “value buyer” to “strategic buyer”

As the single largest payer, the NHSA has claimed the role of “strategic buyer” on behalf of local government, providers, and patients. This function is also consistent with China's healthcare reforms, which bring together three “medical forces” (medicines, hospitals, and health insurance). As a strategic buyer, the NHSA may take into account its significant influence over the healthcare sector and develop unique market mechanisms to foster competition and advances in high‐quality and affordable drugs. The industry's management across the product lifecycle, including time‐to‐market, price, and marketing strategy, will be affected by the NHSA's buying strategies.

#### 3.3.4 Innovative payment for innovations

Innovative payment methods such as risk‐sharing market access agreements (finance‐based and performance‐based), and some novel funding models for expensive therapies (annuity/installment payments) have gained policy attention in China in recent years. For example, some commercial insurance carriers have started experimenting with outcome‐based agreements with drug companies for a limited range of CAR‐T treatments. Looking forward, some true innovations that are critical to treatment may temporarily be ineligible for NRDL reimbursement or need more time to build evidence of their value. It may then be worth considering innovative payment schemes. Coverage with evidence development (CED), an internationally adopted alternative reimbursement model, could be leveraged to accelerate access to innovative drugs in the future.

It will not be easy to balance medical innovation and affordability to achieve a win–win solution in China. However, the ongoing healthcare reforms and efforts from the central payer to provide innovative payment methods to accommodate emerging drug development and advanced therapies suggest that there is willingness to at least work toward this aim.

## AUTHOR CONTRIBUTIONS

*Writing original draft and project management*: Vivian Chen. *Validation and writing reviewing and editing*: Wenbin Shao. *Equal*: all others.

## CONFLICT OF INTEREST

The authors declare no conflict of interest.

## ETHICS STATEMENT

Ethics statement is not applicable to this study.

## INFORMED CONSENT

None.

## Data Availability

The data that support the findings of this study are available from the corresponding author upon reasonable request.
